# Anatomy, histochemistry, and immunohistochemistry of the olfactory subsystems in mice

**DOI:** 10.3389/fnana.2014.00063

**Published:** 2014-07-14

**Authors:** Arthur W. Barrios, Gonzalo Núñez, Pablo Sánchez Quinteiro, Ignacio Salazar

**Affiliations:** ^1^Unit of Anatomy and Embryology, Department of Anatomy and Animal Production, Faculty of Veterinary, University of Santiago de CompostelaLugo, Spain; ^2^ICT Department, Hospital PolusaLugo, Spain

**Keywords:** nasal cavity, morphology, digital atlas, olfactory epithelium, subdivisions, mouse

## Abstract

The four regions of the murine nasal cavity featuring olfactory neurons were studied anatomically and by labeling with lectins and relevant antibodies with a view to establishing criteria for the identification of olfactory subsystems that are readily applicable to other mammals. In the main olfactory epithelium and the septal organ the olfactory sensory neurons (OSNs) are embedded in quasi-stratified columnar epithelium; vomeronasal OSNs are embedded in epithelium lining the medial interior wall of the vomeronasal duct and do not make contact with the mucosa of the main nasal cavity; and in Grüneberg's ganglion a small isolated population of OSNs lies adjacent to, but not within, the epithelium. With the exception of Grüneberg's ganglion, all the tissues expressing olfactory marker protein (OMP) (the above four nasal territories, the vomeronasal and main olfactory nerves, and the main and accessory olfactory bulbs) are also labeled by *Lycopersicum esculentum* agglutinin, while *Ulex europaeus* agglutinin I labels all and only tissues expressing G_αi2_ (the apical sensory neurons of the vomeronasal organ, their axons, and their glomerular destinations in the anterior accessory olfactory bulb). These staining patterns of UEA-I and LEA may facilitate the characterization of olfactory anatomy in other species. A 710-section atlas of the anatomy of the murine nasal cavity has been made available on line.

## Introduction

Though between-species differences are notorious, the olfactory systems of mammals are in general able to recognize thousands of odorant molecules that vary in shape, size, charge, and function. These molecules are captured by the odorant receptors that populate the terminal dendritic trees of olfactory sensory neurons (OSNs) located in up to four areas of the epithelium lining the nasal cavities (Buck and Axel, 1991; Buck, 1996). The major such area is the main olfactory sensory epithelium (MOE) (Graziadei, 1971); the other nasal structures with OSN-bearing epithelium that may be present are the vomeronasal organ (VNO) (Jacobson, 1813; Doving and Trotier, 1998; Zancanaro, 2014), the septal organ (SO) (Broman, 1921; Rodolfo-Masera, 1943), and the ganglion of Grüneberg (GG) (Grüneberg, 1973). All these territories have been identified in mice (Breer et al., 2006; Storan and Key, 2006).

Of the four territories mentioned above, the GG was largely overlooked until its “rediscovery” a decade ago (Fuss et al., 2005; Koos and Fraser, 2005; Roppolo et al., 2006; Storan and Key, 2006), since when it has attracted considerable interest (Brechbühl et al., 2008; Schmid et al., 2010; Mamasuew et al., 2011; Matsuo et al., 2012). The other three have often been hypothesized as having distinct olfactory functions, an idea that in the case of the VNO and MOE is supported by the fact that whereas MOE OSNs project to the main olfactory bulb (MOB), the OSNs of the sensory epithelium of the VNO (the VNsE) project to the accessory olfactory bulb (AOB). Indeed, in both these cases more detailed correspondences have been distinguished: the apical and basal regions of the VNsE project to the anterior and posterior AOB, respectively (Jia and Halpern, 1996; Salazar and Sánchez-Quinteiro, 2003), while four MOE zones have been reported to correspond to four MOB regions defined along an anterodorsomedial-caudoventrolateral axis (Ressler et al., 1993; Vassar et al., 1993). However, more exact MOE-MOB relationships are based on types of OSN rather than the MOE area they occupy (Munger et al., 2009; Ma, 2010; Mori and Sakano, 2011; Murthy, 2011); and, more importantly, the anatomical and functional independence of the vomeronasal and main olfactory systems is questioned by a number of findings (Boehm et al., 2005; Mandiyan et al., 2005; Yoon et al., 2005), notably the feedback from higher centers. Much remains to be known about the interactions of these two systems with each other (Keverne, 2005; Brennan and Zufall, 2006; Shepherd, 2006; Mucignat-Caretta et al., 2012) and with the GG and SO (Levai and Strotmann, 2003; Ma et al., 2003; Kaluza et al., 2004; Tian and Ma, 2008).

In view of the above, the MOE, VNsE, SO and GG can be considered as the entry points of four olfactory subsystems (OSbS), the integration of which at higher levels has yet to be determined. To consolidate and possibly refine the structural basis of this approach in a way that would be readily applicable to other mammals, in the work described here we documented the morphology of the entire nasal cavity of the mouse and studied selected territories histochemically (using the lectins *Ulex europaeus* agglutinin-I and *Lycopersicum esculentum* agglutinin) and immunohistochemically [using antibodies against olfactory marker protein (OMP) and the G-protein subunits G_αi2_ and G_α0_]. OMP is considered to be a marker of all olfactory neurons; G_αi2_ and G_α0_ differentiate vomeronasal OSNs projecting to different AOB territories (Wekesa and Anholt, 1999); and the two lectins used have coherent staining patterns in the olfactory bulbs (Salazar et al., 2001).

Our morphological material is available on-line as a 710-section digital atlas of the murine nasal cavity (see Supplementary Material below). As far as we know, this is the first time that the anatomies of all four olfactory territories have been presented together in relation to the cavity as a whole.

## Materials and methods

### Animals

Fourteen male or female healthy BALB/c mice aged at least 10 months, reared in the animal care facilities of the University of Santiago de Compostela (Registry No. 15003AE), were euthanized and decapitated in the Department of Pharmacology for use as control animals in pharmacological research; housing and handling followed the guidelines of the USC Biethical Committee. The intact heads were kindly donated to the authors.

### Processing of samples and tissue sections

Eight heads were fixed by immersion in 10% buffered formalin (Fr) and stored in 4% Fr. The other six heads were fixed by immersion in Bouin's fixative (Bn), and after 24 h were transferred to 70% alcohol.

For examination of the entire nasal cavity, two Fr-fixed heads were decalcified in Shandon TBD-1 rapid decalcifier (Thermo, Pittsburgh, PA), oriented so that the hard palate was horizontal, embedded in paraffin wax, and cut in transverse sections 8-10 μm thick perpendicular to the hard palate. Alternate sections (710) were transferred to slides and stained with haematoxylin-eosin (HE).

From two Fr- and two Bn-fixed heads, the brain was removed and transferred again to Fr or Bn. The remaining pieces of the these heads, and the other eight heads (four Fr- and four Bn-fixed), were decalcified, oriented as above, and embedded, and serially cut transverse sections were transferred to slides. In the light of the information obtained from the entire nasal cavity series (see above), selected sections at seven different levels intersecting the GG, VNO, and SO, and at four levels of the posterior MOE, were stained with HE, and the remainder were used in histochemical and immunohistochemical protocols.

Fr- and Bn-fixed olfactory bulbs were used as controls for the histochemical and immunohistochemical procedures described below.

### Lectin histochemistry

The lectins *Ulex europaeus* agglutinin I (UEA-I) and *Lycopersicum esculentum* agglutinin (LEA) were obtained as biotin conjugates from Sigma (St. Louis, MO, USA). Tissue sections were (1) incubated for 30 min at room temperature with 2% bovine serum albumin in 0.1 M phosphate buffer (PB, pH 7.2); (2) incubated for 24 h at 4°C with lectin at concentrations ranging from 20 to 60 μg/mL in 0.1 M Tris buffer containing 0.5% bovine serum albumin; (3) washed for 2 × 10 min in PB; (4) incubated for 90 min at room temperature with Vectastain ABC reagent (1:250 in PB); and (5) incubated in 0.2 M Tris–HCl buffer (pH 7.6) containing 0.05% 3,3-diaminobenzidine and 0.003% H_2_O_2_. Controls were run without lectin or with preabsorption of lectin by an excess amount of the corresponding sugar.

### Immunohistochemistry

Immunohistochemical studies were performed using antibodies against olfactory marker protein (OMP) (Wako Chemicals, 1:500 dilution) and the G-proteins G_αi2_ (Santa Cruz Biotechnology, 1:100) and G_α0_ (Santa Cruz Biotechnology and Medical & Biological Lab Co., 1:100). Sections were dewaxed in xylene, rehydrated, and successively incubated (1) for 30 min at room temperature in PB containing 5% normal horse serum and 2% bovine serum albumin, (2) for 24 h at 4°C in primary antibody solution, (3) for 1 h in biotinylated secondary antibody solution, and (4) for 2 h in a solution of avidin-biotin-horseradish peroxidase complex (ABC Vectastain reagent); after which standard procedures for visualization of the horseradish peroxidase complex with 3,3-diaminobenzidine were followed, and the sections were dehydrated through alcohols, cleared in xylene, and coverslipped.

### Image acquisition and processing

Digital images were captured using a Karl Zeiss Axiocam MRc5 digital camera. When necessary, Adobe Photoshop CS4 (Adobe Systems, San Jose, CA) was used to adjust contrast and brightness to equilibrate light levels, and/or to crop, resize and rotate the images for presentation; no additional digital image manipulation was performed.

### Implementation of the on-line atlas (supplementary material)

The on-line atlas was developed using the languages HTML5, CSS3, JavaScript, PHP5 and XML, the libraries/frameworks JavaScript jQuery (v1.10.1) and Normalize.css (v2.1.3), and the scripts/plug-ins PhpThumb (v3.0), JQuery Sketch.js, Zoomooz (v1.1.6), ColorBox (v1.3.31), waitForImages, FitText (v1.1), qTip2 (v2.1.1), and Image Power Zoomer (v1.1). It consists of a front end comprising the content and associated functionality (accessible in any current web browser, including the latest versions of Mozilla Firefox, Google Chrome, Opera, Safari and Internet Explorer) and a back end PHP-based area for merging and managing the data, images and texts.

## Results

### Anatomy

**Figure 1:**
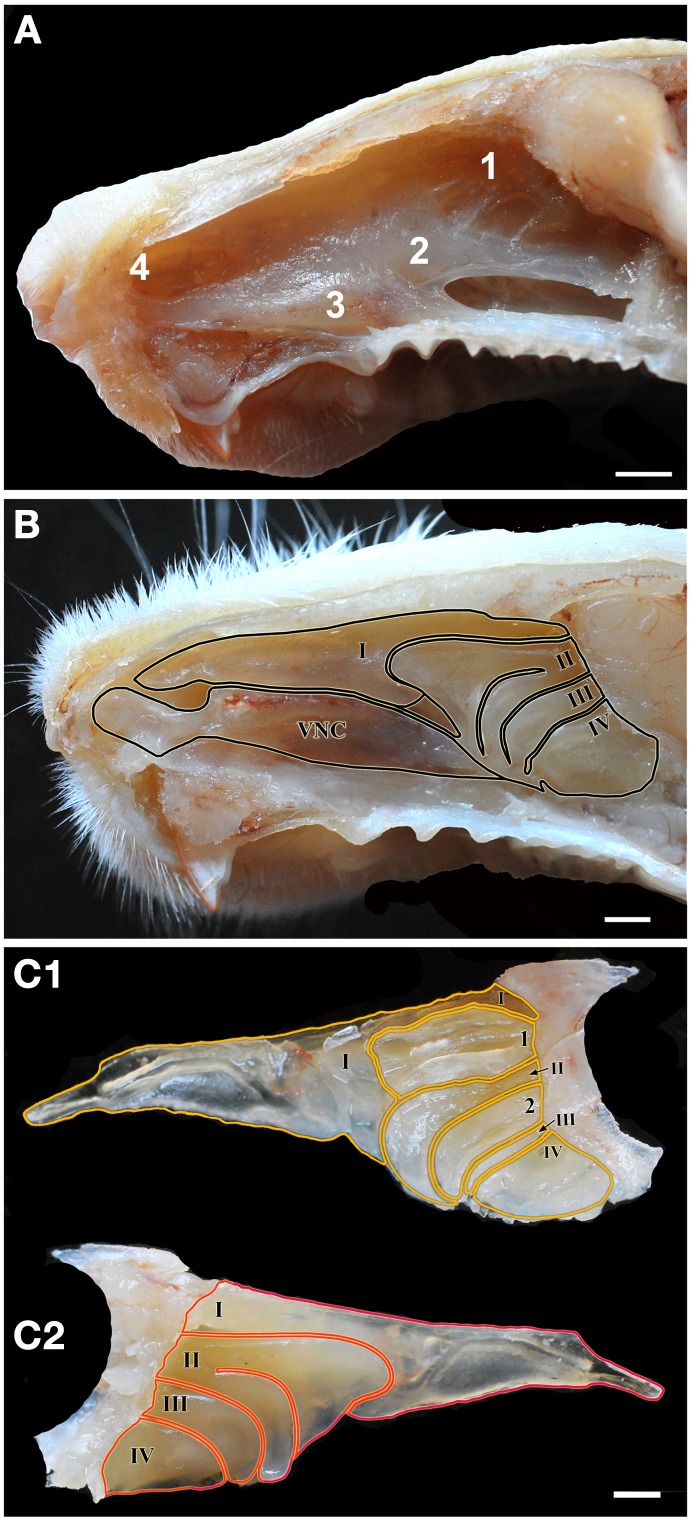
**Views of the nasal cavity. (A)** The nasal septum, showing the location of the main olfactory epithelium (1), the septal organ (2), the vomeronasal organ (3) and Grüneberg's ganglion (4). **(B)** Medial view of the nasal conchae and ethmoturbinates following removal of the nasal septum, with endoturbinates numbered by Roman numerals (VNC, ventral nasal concha). **(C)** Lateral **(C1)** and medial **(C2)** views of the isolated dorsal nasal concha and ethmoturbninates, with ectoturbinates numbered by Arabic numerals and endoturbinates as in **(B)**. Scale bars: **(A)** 2 mm; **(B,C)** 1 mm.

**Figure 2:**
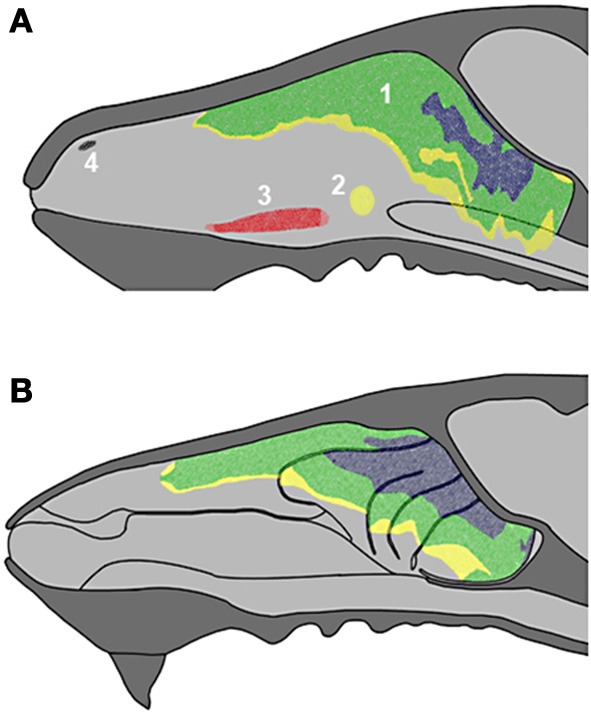
**Schematic drawings of the nasal septum (A) and the medial aspect of the nasal cavity (B), showing the locations of the main olfactory epithelium (1), septal organ (2), vomeronasal organ (3) and Grüneberg ganglion (4) with indication of epithelial thickness (yellow, thin; green, medium; blue, thick).** See supplementary material.

**Figure 3:**
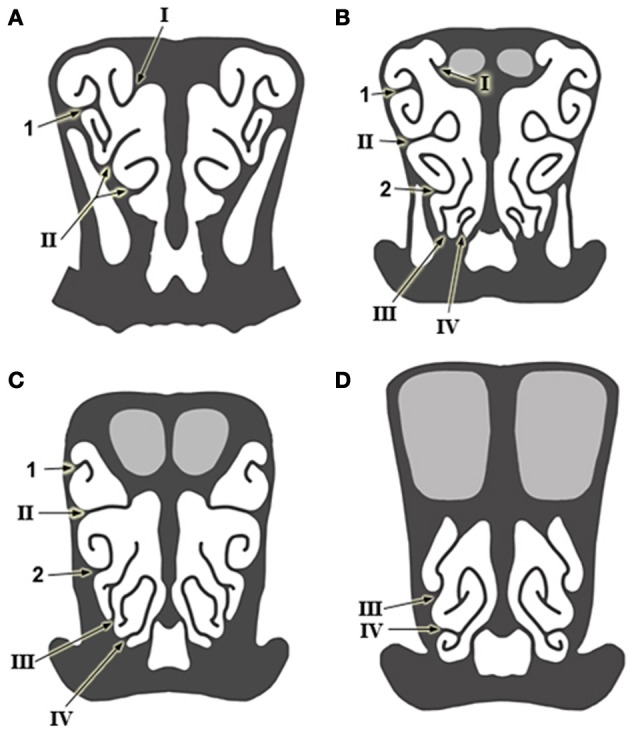
**Schematic drawings of transverse sections of the head, from anterior to posterior levels **(A–D)**, showing the arrangement of the ethmoturbinates at several levels in the posterior part of the nasal cavity.** Numbering as in Figure 1.

Figures 1A, 2 (see also the Supplementary Material) show the locations of the MOE, SO, VNO and GG, which occupy the mucosal lining of most of the nasal cavity except the ventral concha (for turbinate numbering, see Figures 1, 3).

**Figure 4:**
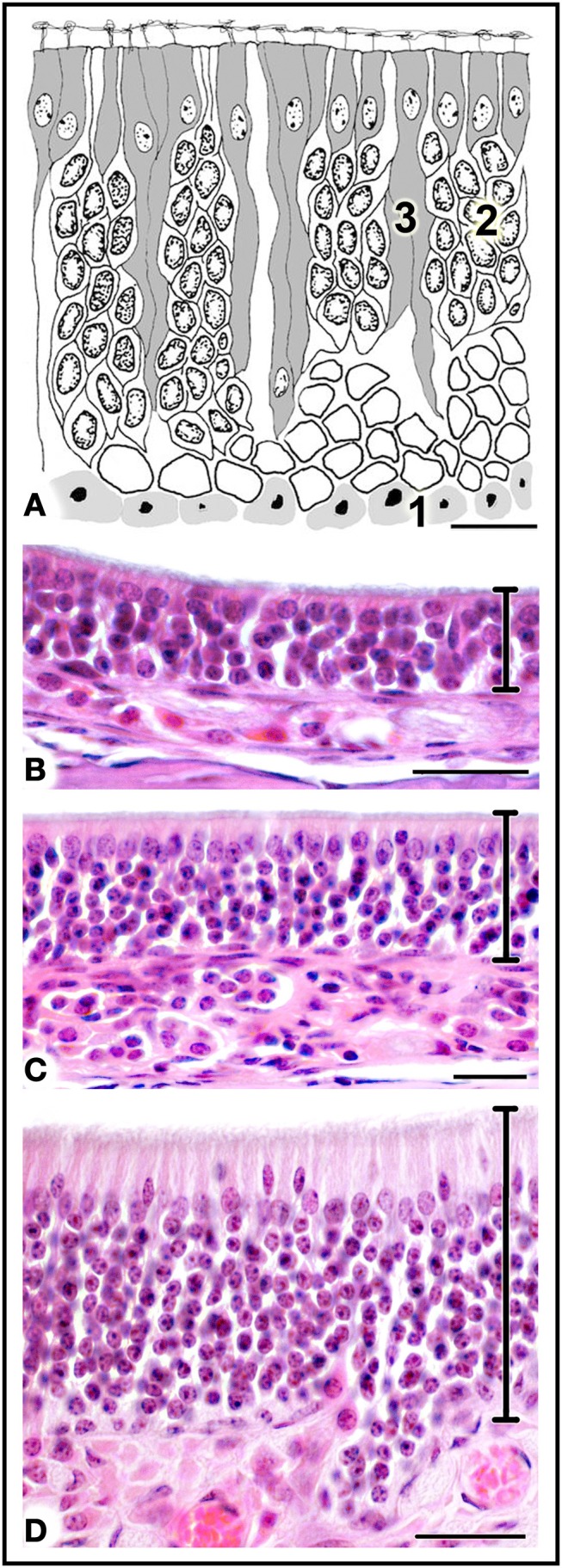
**(A)** Diagrammatic reconstruction of main olfactory epithelium, showing basal cells (1), mature neurons (2) and supporting cells (3) (modified after Graziadei, 1971). **(B,C)** Haematoxylin-eosin-stained sections of areas of epithelium with different thicknesses (see text). Scale bars: **(A)** 10 μm; **(B)** 20 μm; **(C,D)** 25 μm.

**Figure 5:**
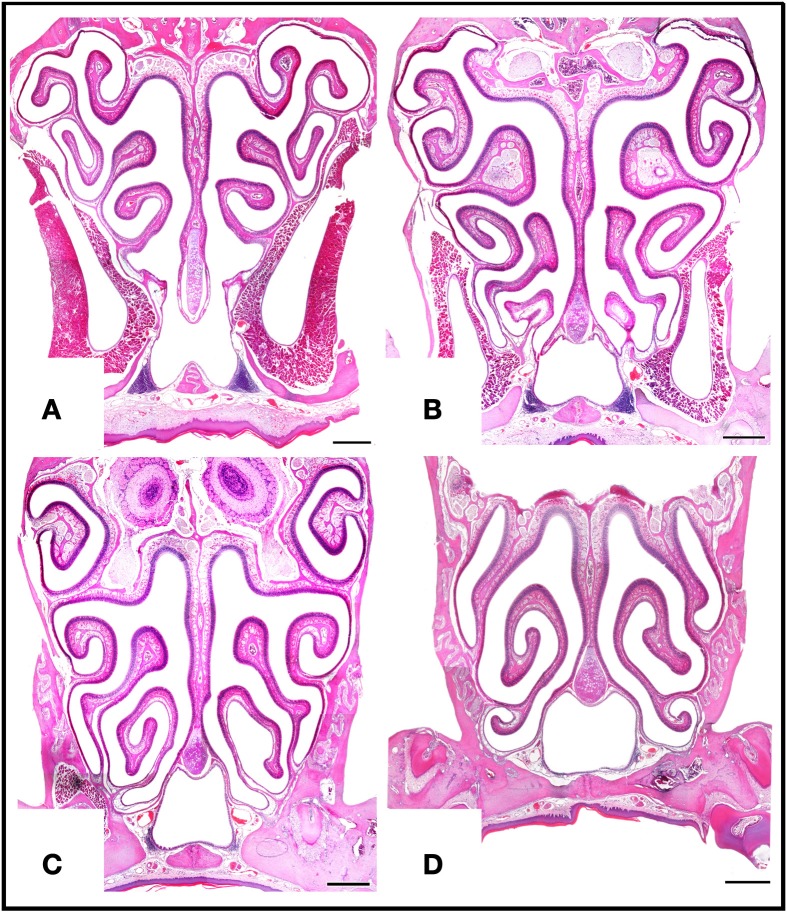
**Haematoxylin-eosin-stained transverse sections at four levels of the posterior nasal cavity, from anterior to posterior levels **(A–D)**, where most of the MOE is located.** Scale bars: 500 μm.

The MOE features three major cell types: neurons, supporting cells, and the basal stem cells that generate olfactory neurons throughout life (Figure 4A). Three regions may be distinguished on the basis of whether the MOE tissue is locally, on average, (i) 3–5, (ii) 6–10, or (iii) 11 or more cells thick (Figures 4B–D, 5). In general, the MOE is thicker in dorsal regions than in the corresponding ventral regions, and there are similar thick-thin differences in the medial-lateral and posterior-anterior directions (see Supplementary Material).

**Figure 6:**
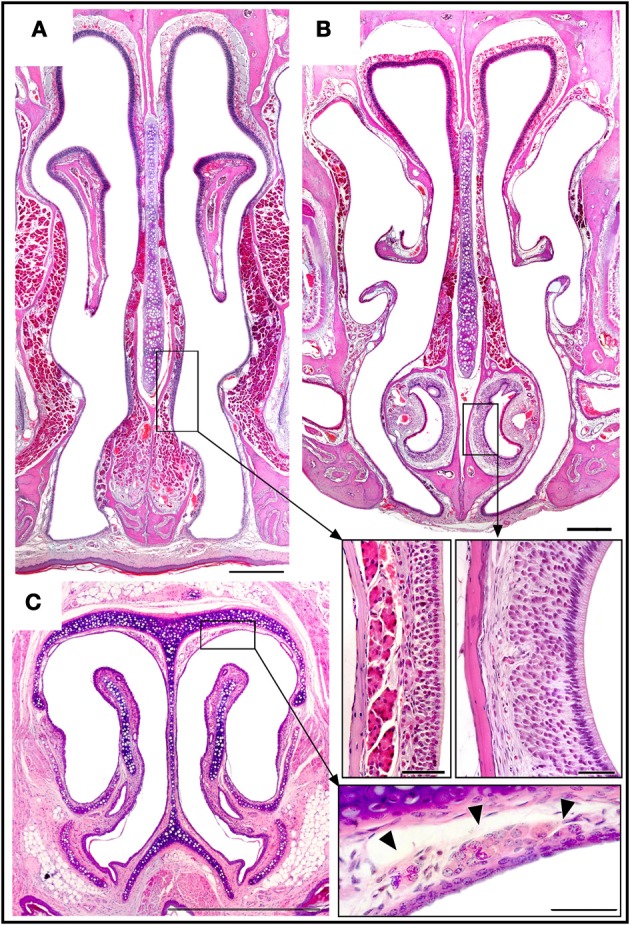
**Haematoxylin-eosin-stained transverse sections showing the locations of the septal organ (A), the vomeronasal organ (B), and Grüneberg's ganglion (C), together with an enlarged view of each in the corresponding inset.** Topography of the rüneberg's ganglion cells (arrows heads). Scale bars: **(A–C)**, 500 μm; insets, 50 μm.

The SO is an independent structure with the same characteristics as MOE region (i) (Figure 6A).

The VNO occupies a thin cylindrical lamina of bone located on the floor of the nasal cavity adjacent to the vomer. It comprises the vomeronasal duct (a blind epithelial tube with a single small rostral orifice connecting it with the main nasal cavity) together with surrounding glands, vessels, nerves, and connective tissue. The VNsE is limited to the central levels of the medial wall of the duct (Figure 6B).

In the GG, located in the nasal vestibule adjacent to the nasal septum and the dorsolateral nasal cartilage (Figure 6C), a small isolated group of OSNs are embedded in connective tissue under a dense network of blood vessels, adjoining but not within the epithelium. Among adult mice there is significant between-individual variation in GG anatomy; for example, symmetry between the right and left sides is not universal.

### Histochemistry and immunohistochemistry

#### Main olfactory epithelium

**Figure 7:**
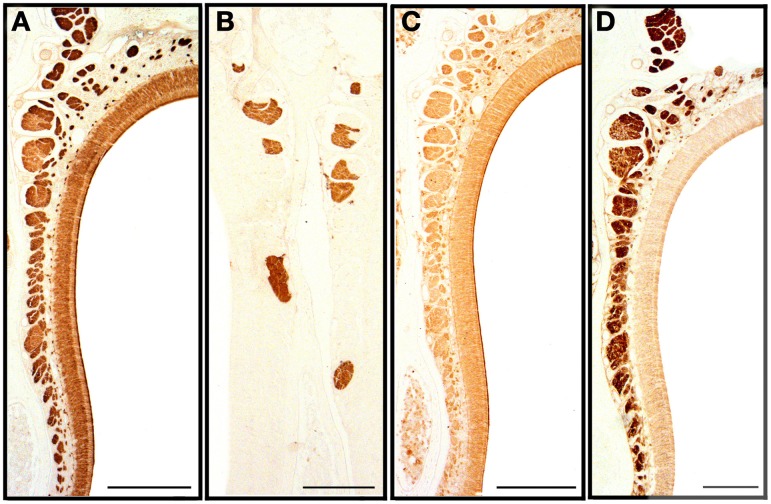
**Transverse sections of the nasal septum stained with anti-OMP (A), UEA-I (B), LEA (C) and anti-G_α0_ (D), showing the olfactory sensory epithelium and the olfactory and vomeronasal nerve bundles**. Scale bars: **(A,C)** 150 μm; **(B,D)** 100 μm.

**Figure 8:**
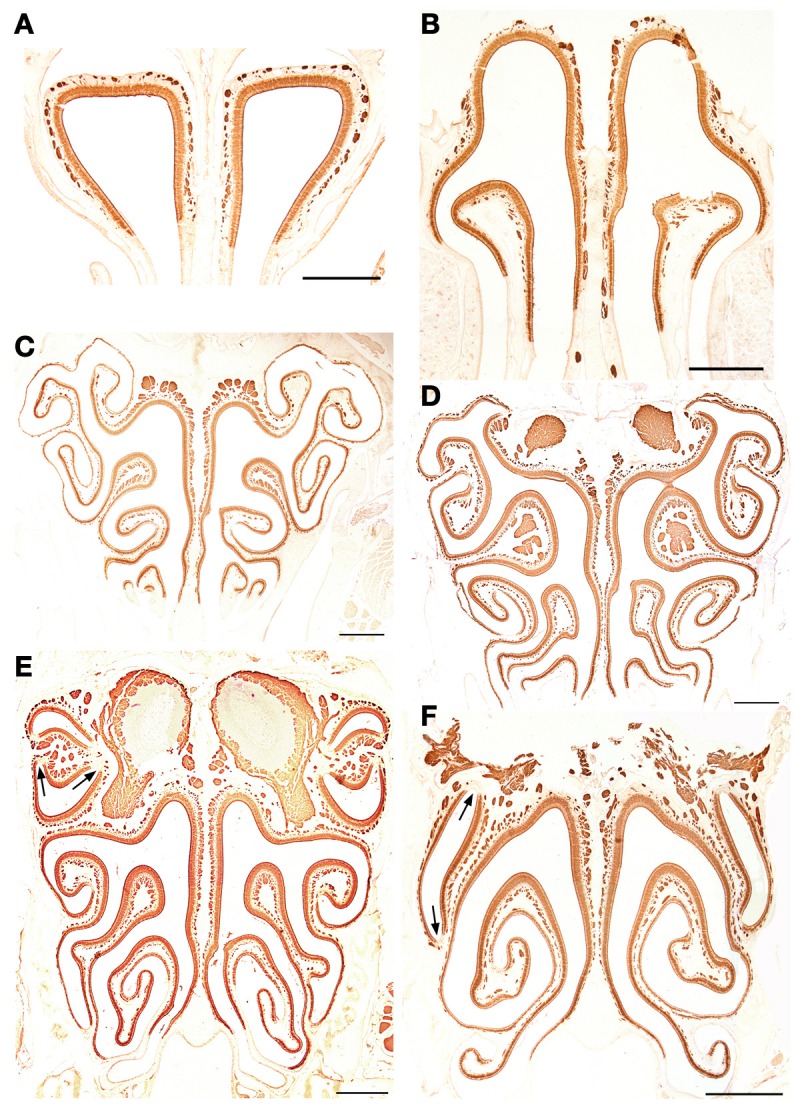
**Anti-OMP-stained transverse sections of the nasal cavity at the central levels of the vomeronasal organ (A) and septal organ (B), and at the same levels as in Figure 4 (C–F).** Note the similar reactivities of epithelium and axon bundles. In **(E,F)**, arrows indicate small areas devoid of immunoreactivity. Scale bars: 500 μm.

**Figure 9:**
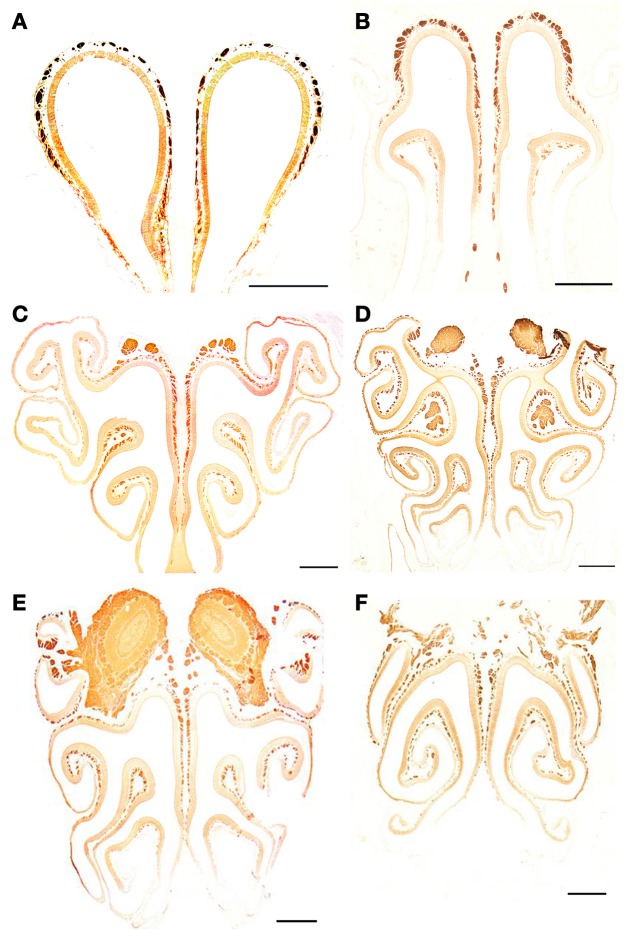
**Anti-G_α0_-stained transverse sections of the nasal cavity at the same levels **(A–F)** as in Figure 8**. Note the different reactivities of epithelium and axon bundles. Scale bars: 500 μm.

We first applied all the histochemical and immunohistochemical stains to sections in which both main olfactory and vomeronasal nerves run adjacent to the septum. Though with different intensities, anti-OMP and LEA stained the MOE and both the main olfactory and vomeronasal nerves bundles (NBo and NBv, respectively); anti-G_α0_ strongly labeled all nerve bundles; and UEA-I and anti-G_αi2_ bound only to NBv (Figure 7). The results of staining for OMP (Figure 8) and G_α0_ (Figure 9) at six different transverse levels of the nasal cavity were in agreement with these findings.

#### Vomeronasal organ, septal organ, and the ganglion of Grüneberg

**Figure 10:**
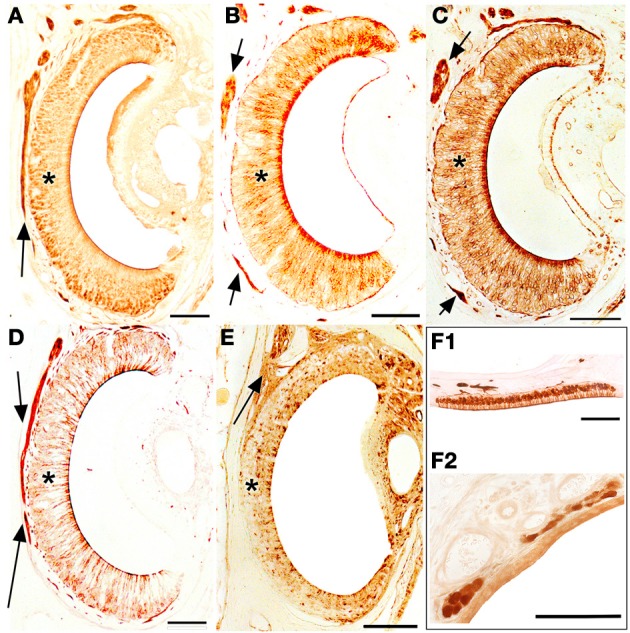
**Transverse sections of the vomeronasal duct stained with anti-OMP (A), UEA-I (B), LEA (C), anti-G_α0_ (D) and anti-G_α*i*2_ (E), and of the septal organ (F_1_) and Grüneberg ganglion (F_2_) stained with anti-OMP**. Arrows indicate vomeronasal nerve axons medial to the vomeronasal sensory epithelium (asterisk). Scale bars: 100 μm.

The staining behavior of UEA-I, anti-G_α0_ and anti-G_αi2_ in the VNsE distinguished apical and basal layers, UEA-I and anti-G_αi2_ staining the apical VNsE (VNa), and anti-G_α0_ the basal VNsE (VNb) (in this case mainly at the edges of the sensory epithelium) (Figures 10B,D,E). Anti-OMP and LEA stained both layers (Figures 10A,C). The staining pattern of the SO was essentially identical to that of the MOE (Figure 10F1 shows OMP positivity in an SO section). GG cells were also clearly OMP-positive, but accepted no other stain (Figure 10F2).

#### Olfactory bulbs

**Figure 11:**
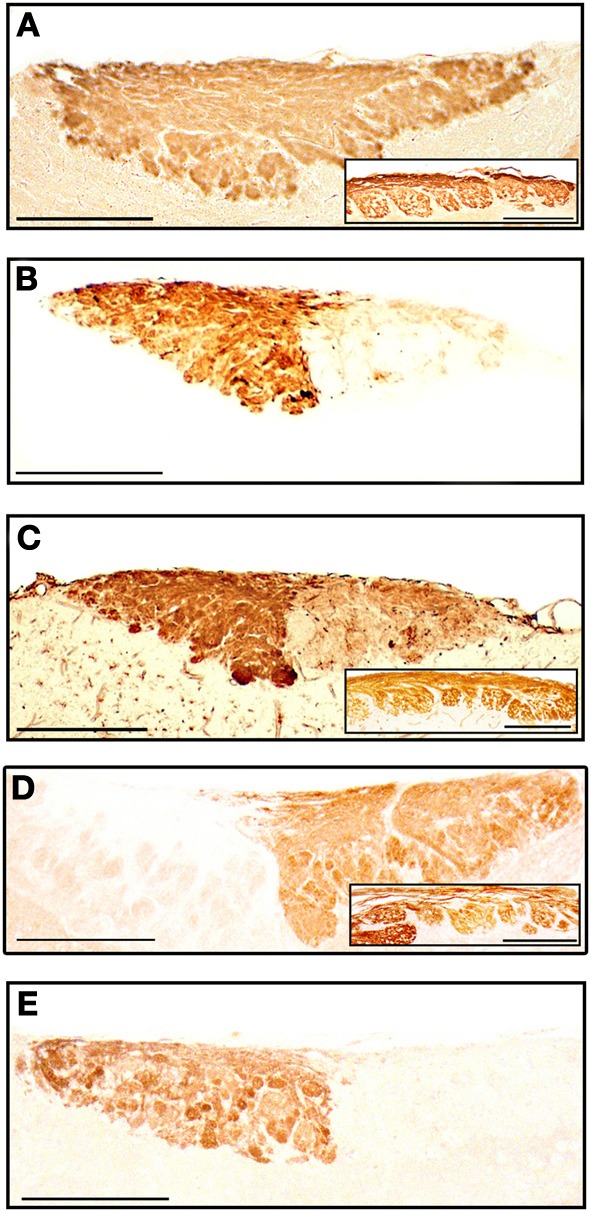
**Parasagittal sections of the olfactory bulb through the AOB (left anterior, right posterior) stained with anti-OMP (A), UEA-I (B), LEA (C), anti-G_α0_ (D) and anti-G_α*i*2_ (E)**. Insets show the nervous and glomerular layers of the MOB. Scale bars: **(A)** 250 μm; **(B–E)** and insets, 200 μm.

The staining patterns of the olfactory bulbs used as controls are shown in Figure 11. Note that like anti-OMP, LEA stains both the nervous and glomerular layers of both the MOB and the AOB.

## Discussion

The above results clearly identify four different olfactory sensory areas in the nasal cavities, and accordingly justify the use of OSbS terminology. In regard to the anatomy of the VNO, it is perhaps worth stressing that vomeronasal OSNs do not make contact with the mucosa of the main nasal cavity. For them to bind the semiochemicals to which they respond, these latter must therefore be pumped into the vomeronasal duct in some way, probably by vascular constriction (Meredith et al., 1980; Salazar et al., 2008). Thus, the non-epithelial components of the VNO do not merely provide mechanical and physiological support for the sensory epithelium, but must play an active role in olfaction. This raises a question as to the triggering of this pumping mechanism, which if vascular is presumably not of itself a voluntary action. One possibility is that it may be part of some general scent-seeking behavioral pattern; another, that it may be triggered by the detection of an olfactory signal of broad significance by one of the three olfactory territories of the main nasal cavity.

The possibility of further defining subdivisions of the olfactory epithelial territories on the basis of the receptor types borne by OSNs appears to depend on both the OSbS and the broad class of receptor types in question. The VNsE is clearly divisible into an apical stratum with OSNs bearing receptors of type V1R, and a basal stratum with V2R-bearing OSNs (Wagner et al., 2006). The axons of these apical and basal strata respectively project to the anterior and posterior regions of the AOB. Further, the upper and lower sublayers of the basal VNsE respectively project to the anterior and posterior parts of the posterior AOB, in correlation with whether the V2R OSNs do not or do express *H2-mv* genes (Salazar and Sánchez-Quinteiro, 2003; Ishii and Mombaerts, 2008). However, these divisions appear to be crossed by OSNs bearing formyl peptide receptors, which seem to be widely dispersed in either the entire VNsE (Liberles et al., 2009) or, with a rostrocaudal gradient that is more pronounced in juveniles than adults, in its apical layer (Rivière et al., 2009; Dietschi et al., 2013). Moreover, the functional significance of these subdivisions is unknown, and its elucidation will require more extensive investigation of the ligands recognized by the vomeronasal system (Chamero et al., 2012; Francia et al., 2014).

In the MOE, the first reports of zonal organization of OSNs bearing the multiple varieties of “canonical” G-protein-coupled olfactory receptor (OR), made possible by the cloning of odorant receptor genes (Buck and Axel, 1991), spoke of each receptor-defined OSN type being distributed at random in just one of three or four zones (Ressler et al., 1993; Vassar et al., 1993). Subsequently, a more refined scheme emerged that related to the distinction between phylogenetically older (class I) and younger (class II) OR types: while almost all OSNs with a class I OR type or belonging to a subset of class II OR types are randomly located in a dorsomedial region (D), each of the multiple other class II types is borne exclusively by OSNs occupying a type-specific antero-posterior swathe in the remainder of the MOE (V), with the centerline of one swathe displaced dorsoventrally just slightly from that of the next, so that each swathe overlaps multiple others (Miyamichi et al., 2005). The projection of the OSNs defined by a given OR type to just a single pair of MOB glomeruli, confirmed by experiments with P2-IRES-tau-lacZ mice (Mombaerts et al., 1996), makes the detailed map of the MOE in the MOB discrete (Luo and Flanagan, 2007), but this map nevertheless respects the zonal organization described above: OR OSNs in MOE region D project to the dorsal MOB (Kobayakawa et al., 2007; Bozza et al., 2009), and the dorsoventral order of OR types in region V is faithfully reproduced by the glomeruli to which their OSNs project in the ventral MOB. Also, the more recently discovered subset of OR OSNs that express TRPM5, which are mainly located in the ventrolateral MOE, mainly project to glomeruli in the ventral MOB (Lin et al., 2007); and a similar degree of topographical correspondence is seen in regard to OSNs bearing trace-amine-associated receptors (TAARs, one of the two known types of non-OR olfactory receptor in the MOE; Liberles and Buck, 2006), TAAR OSNs located in the dorsal MOE projecting to two or three glomeruli in a well-defined dorsal area of the MOB (Pacifico et al., 2012). However, topographicness is less evident in the mapping of OSNs with guanylyl cyclase D receptors, the other non-OR MOE receptor type. These OSNs, which are found in clusters in a limited MOE zone (mostly in the dorsal recesses of the nasal cavity), project to a number of the “necklace glomeruli” surrounding the caudal end of the MOB (Gibson and Garbers, 2000; Walz et al., 2007).

In the present study we found that the MOE is in general thicker in the dorsal zone than the ventral, thicker in the medial zone than the lateral, and thicker in the posterior zone than the anterior. The relevance of these findings to the zonal organization described above is unclear, but they should be taken into account in future work, among other reasons because they are consistent with inspiratory airflow paths (Schoenfeld and Cleland, 2005).

The olfactory bulbs were initially included in this study merely as control tissues, since in previous work they have reacted very well with the lectins employed in the present study, exhibiting consistent, anatomically coherent patterns: although staining uniformity depends upon the age of the animal (Salazar and Sánchez-Quinteiro, 2003), LEA labels the glomeruli and incoming nerves of both the AOB and the MOB, while UEA-I binds only to the vomeronasal nerves and AOB structures (Salazar et al., 2001). Our present results confirm these findings and extend them to the corresponding sensory epithelia. UEA-I thus emulates anti-G_αi2_ in its specificity (under the conditions of this study) for the olfactory subsystem entered via the apical VNsE; while LEA, except for its failure to stain the Grüneberg ganglion, emulates the neuron-staining behavior of anti-OMP, which is specific for the olfactory nervous system as a whole (Margolis, 1972) (in Figures 8E,F, note the absence of stain in acute angles of the turbinates, where neural cells are lacking; Suzuki et al., 2000). Although LEA, unlike anti-OMP, also stains glands, vessels and other tissues adjoining neurons, and is also prone to non-specific staining, this mimicry of anti-OMP by LEA and of anti-G_αi2_ by UEA-I, which is also found in a number of other species (see Salazar and Sánchez-Quinteiro, 2009), allows the use of these inexpensive lectins to obtain prima facie evidence concerning the structure of the VNsE in hitherto unstudied species. Although the molecular basis of this mimicry, i.e., the identity of the sugar-bearing molecules to which UEA-I and LEA bind in the olfactory system, is not totally clear, it appears to involve the role of cell surface blood group antigens in the wiring of the olfactory system (see, for example, St John et al., 2006).

In this study, neither UEA-I nor anti-G_αi2_ bound to the MOE, the septal organ, or Grüneberg's ganglion. The absence of binding to the MOE appears to contradict a report that G_αi2_ is expressed in olfactory neurons located near the dorsal septum and in the dorsal recess of the nasal cavity, and in MOB glomeruli concentrated mainly on the medial side (Wekesa and Anholt, 1999); it seems possible that the neurons stained by Wekesa and Anholt in the nasal cavity may have been vomeronasal nerves, although this would not explain their observations in the MOB. Our results are in keeping with those of Wekesa and Anholt (1999) in that expression of G_α*o*_ was restricted to the basal VNsE and to the glomeruli and incoming axons of the MOB and posterior AOB. That Grüneberg's ganglion was stained by anti-OMP but by neither anti-G_α*o*_ nor anti-G_αi2_ is in keeping with the findings of Roppolo et al. (2006), who observed mRNA for OMP but not for then known ORs, V1Rs or V2Rs, but not with studies that have detected several TAARs (Fleischer et al., 2007) and the novel vomeronasal receptor V2r83 (Fleischer et al., 2006), which is apparently co-expressed with the guanylyl cyclase GC-G (Matsuo et al., 2012).

## Concluding remarks

The discovery of novel genes involved in the initial stages of olfaction has renewed interest in this sensory system, which is evidently more complex than was previously imagined. Even at the grossest level, it is clear that any division into subsystems must define more than just the main and accessory olfactory systems, and at least three sets of criteria are available for refinement of this earlier conception: (i) division into four subsystems in accordance with the four spatially separated areas of sensory epithelium (MOE, SO, VNsE, and GG); (ii) division as above together with further division in accordance with axon targets, giving six subsystems (MOE OSNs projecting to necklace glomeruli, MOE OSNs projecting to non-necklace glomeruli, the SO, apical VNsE OSNs, basal VNsE OSNs, and GG); (iii) division of OSNs in accordance with their receptor type (which affords subsystems with epithelial domains that are both overlapping and fragmented, but is nevertheless a feasible option). However, there is an often overlooked problem in that the more complex the classification, the more difficult it will be to corroborate in larger species (e.g., if criteria require examination of transgenic animals), and the more difficult it will be to adapt to species with olfactory abilities differing from those of mice (Salazar and Sánchez-Quinteiro, 2009).

We suggest that the five-subsystem classification implicit in Table 1 (MOE-NBo-MOB, SO, VNa-NBv-AOBa, VNb-NBv-AOBp, GG) constitutes a basic scheme, the validity of which for any other species can be easily investigated by the methods described in this paper, and which if necessary can be easily adapted in accordance with the results of these procedures. Although there is no doubt that progress in our understanding of the sense of smell will continue to be driven by molecular-genetic approaches (Axel, 2005), it is equally unquestionable that such studies should be consistent with the anatomy of the explored region (Schoenfeld and Cleland, 2005).

**Table 1:** **Summarizes the histochemical and immunohistochemical results**.

	**OMP**	**UEA-I**	**LEA**	**Gαo**	**Gαi2**
GG	+	−	−	−	−
VNa	+	+	+	−	+
VNb	+	−	+	+	−
SO	+	−	+	−	−
MOE	+	−	+	−	−
NBv	+	+	+	+	+
NBo	+	−	+	+	−
AOBa	+	+	+	−	+
AOBp	+	−	+	+	−
MOB	+	−	+	+	−

*Reactivities of the Grüneberg ganglion (GG), apical vomeronasal sensory epithelium (VNa), basal vomeronasal sensory epithelium (VNb), septal organ (SO), main olfactory epithelium (MOE), vomeronasal nerve bundles (NBv), main olfactory nerve bundles (NBo), anterior accessory olfactory bulb (AOBa), posterior accessory olfactory bulb (AOBp) and main olfactory bulb (MOB) with anti-OMP, UEA-I, LEA, anti-G_αo_ and anti-G_αi2_*.

## Author contributions

Ignacio Salazar designed the research and wrote the paper. Arthur W. Barrios, Gonzalo Núñez and Pablo Sánchez Quinteiro performed the work. Arthur W. Barrios Barrios, Gonzalo Núñez, Pablo Sánchez Quinteiro and Ignacio Salazar analyzed and discussed the data.

### Conflict of interest statement

The authors declare that the research was conducted in the absence of any commercial or financial relationships that could be construed as a potential conflict of interest.

## Supplementary material

The on-line atlas of the murine nasal cavity associated with this manuscript as Supplementary Material is available at http://www.usc.es/anatembriol/. It comprises transverse sections of the whole nasal cavity organized in thirty-five 20-section segments and a final 10-section segment. Clicking on Views and then on Analysis shows, superimposed on a lateral view of the nasal cavity, a grid defining the 31 transverse segments. Clicking on a segment opens a window with thumbnails of its 10 or 20 numbered sections; clicking on a thumbnail brings up the section. The **GG** is located in segments 4 and 5 (sections 0522-0626), the **VNO** in segments 12-20 (sections 1511-2416), the **SO** in segments 21-23 (sections 2531-2724), and the **MOE** in segments 12-35 (sections 1421-4621). The turbinates are located as follows: the **dorsal nasal concha** and **endoturbinate I** in segments 12-30 (sections 1421-3812), **endoturbinate II** in segments 20-31 (sections 2412-3926), **endoturbinate III** in segments 26-32 (sections 3031-4126), **endoturbinate IV** in segments 28-35 (sections 3421-4621), **ectoturbinate 1** in segments 23-31 (sections 2733-3923), and **ectoturbinate 2** in segments 26-31 (sections 3114-4016). Yellow indicates MOE with 3-5 rows of cells, green 6-10 rows, and blue 11 or more rows (see also Figure 2).

## Abbreviations

AOB: accessory olfactory bulb
Bn: Bouin fixative
Fr: buffered formalin fixative
GG: ganglion of Grüneberg
HE: haematoxylin-eosin
LEA: *Lycopersicum esculentum* agglutinin
MOB: main olfactory bulb
MOE: main olfactory epithelium
NBo: main olfactory nerve bundles
NBv: vomeronasal nerve bundles
OMP: olfactory marker protein
OR: olfactory receptor
OSbS: olfactory subsystems
OSNs: olfactory sensory neurons
PB: phosphate buffer
SO: septal organ
TAARs: trace-amine-associated receptors
UEA-I: *Ulex europaeus* agglutinin I
VNa: apical vomeronasal sensory epithelium
VNb: basal vomeronasal sensory epithelium
VNO: vomeronasal organ
VNsE: vomeronasal sensory epithelium.
